# Improving ChatGPT’s Performance in Orthopedics: Opportunities Using the CRISPE Framework

**DOI:** 10.2519/josptmethods.2025.0151

**Published:** 2025-04-28

**Authors:** Mark Vorensky, Daniel Peredo, Richard Ferraro, Emily Paris, Asma Mohammadi, Paul Spano, Smita Rao

**Affiliations:** 1 Department of Rehabilitation and Movement Sciences, School of Health Professions, Rutgers University, Newark, NJ.; 2 Rusk Rehabilitation, NYU Langone Health, New York, NY.; 3 Department of Physical Therapy, Steinhardt School of Culture, Education, and Human Development, New York University, New York, NY.

**Keywords:** artificial intelligence, ChatGPT, large language model, musculoskeletal, natural language processing, orthopedics

## Abstract

ChatGPT has been increasingly used in clinical practice, education, and research. In orthopedic research, ChatGPT’s accuracy in clinical decision-making has been a major concern, with results ranging from 33% to 80% accuracy. Inaccuracies from ChatGPT can be harmful to clinicians, trainees, or patients when responses appear plausible, are trusted, and acted upon. A critical limitation in orthopedic research is the lack of structured prompt engineering, which significantly impacts ChatGPT’s performance. The CRISPE (Capacity/Role, Insight, Statement, Personality, Experiment) framework offers a systematic approach to refining prompts and improving response accuracy. This Viewpoint applies the CRISPE framework to recent orthopedic research and highlights opportunities to optimize prompts in ChatGPT. While research is needed to validate and refine prompt engineering tools in orthopedics, these methods have the potential to enhance the accuracy and reliability of ChatGPT’s responses and serve as valuable tools in orthopedic practice, education, and research.

## Graphical Abstract

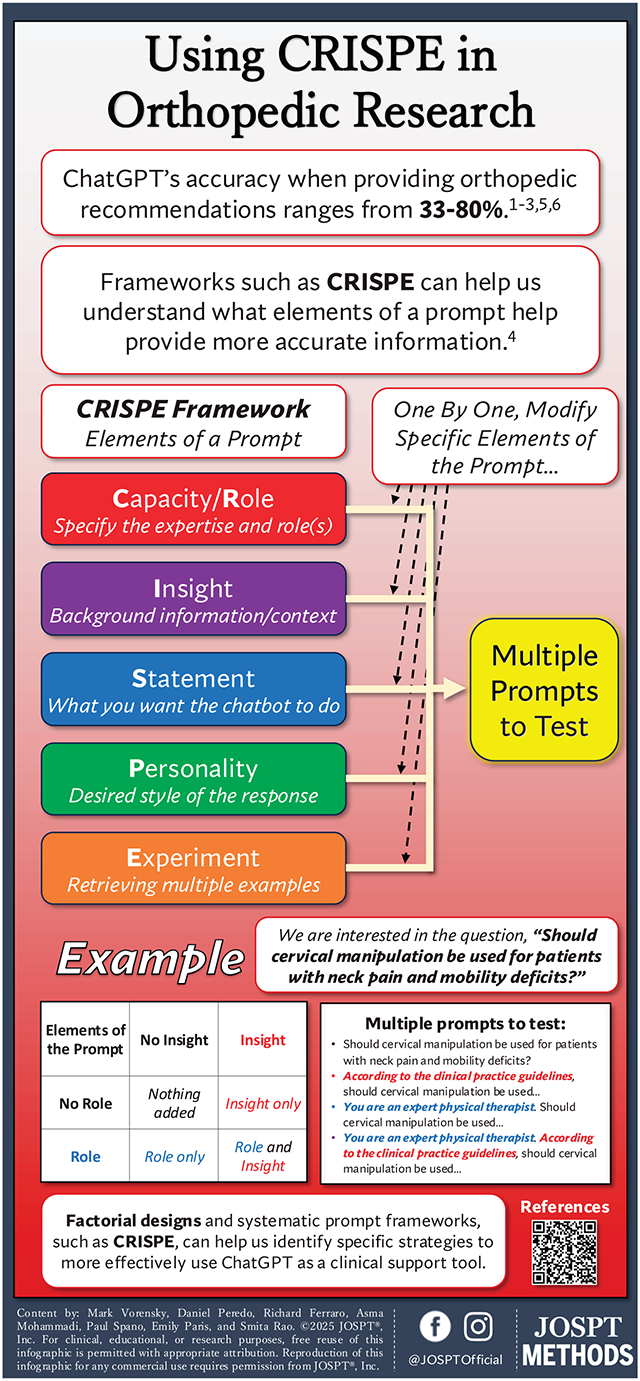

Since its release in 2022, ChatGPT (Chat Generative Pre-trained Transformer), a chatbot from OpenAI, has rapidly become a prominent tool in education, clinical practice, and research. ChatGPT is a product of artificial intelligence (AI), the simulation of human intelligence, performing tasks such as natural language processing, data analysis, and predictive modeling. ChatGPT is built on a large language model, trained on vast data sets, and uses deep learning algorithms to generate human-like text based on input prompts. This model continuously evolves and is fine-tuned as it is exposed to more data, improving its ability to provide relevant and more human-like responses.

Rightful debate surrounds the use of ChatGPT for research, education, and clinical practice. Its quick responses and interactive nature can enhance efficiency; however, concerns include confidentiality, data security, response bias, and diminishing creativity and originality. A major concern surrounds the prevalence of inaccurate and misleading responses, especially when these appear plausible, are trusted, and acted upon.

Orthopedic research has tested ChatGPT accuracy when making clinical decisions.^1,2,4,6,8^ The accuracy of these decisions is often assessed by comparing ChatGPT’s responses to clinical practice guidelines.^2,4,6,8^ This has produced a range of results. The accuracy of clinical decisions for low back pain from ChatGPT-3.5 compared to North American Spine Society clinical practice guidelines was 72%.^8^ For lumbosacral radicular pain, ChatGPT-3.5 showed 33% accuracy compared to multiple clinical practice guidelines.^2^ ChatGPT-4 demonstrated 79% concordance to American Academy of Orthopaedic Surgeons clinical practice guidelines for rotator cuff tears and anterior cruciate ligament injuries.^6^ Similarly, ChatGPT-4 demonstrated 80% accuracy to physical therapy clinical practice guidelines across orthopedic and sports conditions (100% for upper extremity, 60% for spine, 87% for lower extremity conditions).^4^ When assessed by licensed physical therapists, ChatGPT-3.5 did not include elements of physical therapy reassessment and subjective examination 30% and 40% of the time, respectively.^1^ With a wide range of accuracies reported, conclusions are appropriately cautious, such as, “Patients and consumers, who may not have the training to critically evaluate clinical guidelines, should avoid relying on chatbots for musculoskeletal health advice.”^2(p227)^ However, key limitations in current research lead to the question: *Is the problem with ChatGPT or the way we use it?*

## The Primary Limitation of Orthopedic Research Assessing ChatGPT

Single-line prompts are often used when assessing ChatGPT’s accuracy. For example, “Should electrotherapies (such as TENS/PENS/interferential therapy) be used in the management of nonspecific low back pain and sciatica?”^2(p224)^ and “Which are the main steps for a completed physiotherapy assessment?”^1(p2945)^ This highlights a consistent limitation of orthopedic research that assesses ChatGPT’s accuracy, *insufficient prompt engineering*.

Prompt engineering “focuses on developing and optimizing prompts to effectively utilize large language models.”^3(p2629)^ Although prompt engineering is a relatively new area of study, several techniques and frameworks have been proposed.^7,10^ Current research evaluating ChatGPT’s accuracy in providing orthopedic recommendations often do not systematically apply these prompting strategies, potentially leading to variable results.^1,2,4,6,8^ To address these inconsistencies, use of ChatGPT can be optimized through structured prompt frameworks. A promising framework, previously examined in education research, is abbreviated as CRISPE (Capacity/Role, Insight, Statement, Personality, Experiment).^5,9^ The CRISPE framework offers a systematic approach to crafting prompts by defining the following:

- CR: Capacity/Role – specify the expertise and role(s).
- I: Insight – provide background information and context.
- S: Statement – articulate what you would like the chatbot to do.
- P: Personality – indicate the style of the response.
- E: Experiment – retrieve multiple examples.

Examining prompt frameworks, such as CRISPE, has the potential to help researchers understand whether ChatGPT lacks accuracy, if the user is inadequately engineering their prompts, or if there is a combination of both issues. For example, the study by Shrestha et al showed an accuracy for low back pain recommendations that was substantially higher than that of the study by Gianola et al on lumbosacral radicular pain published the same year (72% versus 33%, respectively).^2,8^ This is likely due to additional prompting provided by Shrestha et al, “Imagine you are an experienced orthopedics spine surgeon with a knowledgeable background in the latest research in the field of low back pain. Answer the following prompts based on evidence-based research studies and point out any lack of evidence to support a point (i.e. point out if there is a lack of study to support or refute your answer).”^8(p645)^ Adding this information to the prompt significantly reduced the frequency of insufficient and conflicting responses compared to the question alone.^8^

Excluding the study by Shrestha et al, there has been minimal to no focus on prompt engineering when assessing ChatGPT’s accuracy for orthopedic recommendations. Even in the study by Shrestha et al, it is unclear as to what specific parts of their additional prompt reduced insufficient or conflicting responses. Was it the addition of asking for “evidence-based research” or was it the role in the prompt, “an experienced orthopedics spine surgeon”?^8(p645)^ Systematic approaches that rigorously evaluate details of a prompt are needed to fully understand how to retrieve accurate and reliable information. This limitation opens a huge window of opportunity for orthopedic research, spanning from how prompts are effectively constructed to the ways patients and providers use prompting strategies. Next, we will share an example of how orthopedic researchers may consider prompt engineering in research on the accuracy of ChatGPT.

## Systematically Assessing the Accuracy of ChatGPT

For this example, we will revisit the question from Gianola et al, “Should electrotherapies (such as TENS/PENS/ interferential therapy) be used in the management of nonspecific low back pain and sciatica?”^2(p224)^ Gianola et al found that ChatGPT was inaccurate in its recommendations for this question; however, prompt engineering can be used to understand how to advance ChatGPT’s accuracy. First, the prompt above could be varied across the Capacity/Role, Insight, Statement, Personality, and/or Experiment:

- CR: You are an expert physical therapist
- I: Using clinical practice guidelines.
- S: Should electrotherapies (such as transcutaneous electrical nerve stimulation/percutaneous electrical nerve stimulation/interferential therapy) be used in the management of nonspecific low back pain and sciatica?
- P: Respond as if you are training a physical therapist.
- E: Run the search multiple times.

For a structured and systematic approach, it is recommended that researchers modify 1 factor at a time to examine what element(s) alter ChatGPT’s accuracy. For example, the TABLE and the attached infographic show 2×2 factorial designs that have the potential to determine if explicitly defining the role of ChatGPT and/or adding insight to clinical practice guidelines improves accuracy. There are a range of research opportunities using this methodology, including evaluating how different factors within a prompt affect ChatGPT’s responses and how factors within a prompt interact with each other.

An important consideration is testing prompts multiple times, the “E” of CRISPE. Because ChatGPT considers prior prompts, we recommend that between each search, all prior chats are deleted (in General Settings) and ChatGPT’s memory is cleared (in Personalization Settings). Conducting repeated trials and analyzing variability in responses can help assess inherent model limitations and/or inconsistencies.

## Future Directions

Given the widespread use of ChatGPT among patients, clinicians, trainees, and researchers, a deeper understanding of prompt development and optimization is critical to promote accurate and reliable responses. Research should systematically assess the influence of individual prompt components, such as the role designation, detail of background information, how the core statement is phrased, and the personality of responses. Comparative analyses of different prompt engineering frameworks could also be performed (eg, CRISPE vs CREATE [Context, Result, Explanation, Audience, Tone, and Edit]).^7^ Additionally, there may be complementary prompt techniques, such as retrieval-augmented generation (ie, integrating external databases or evidence within prompts), that may enhance existing frameworks.^10^ Whether studies examine elements of a prompt or compare frameworks, researchers should document and standardize testing conditions, including the version of ChatGPT used, timing of queries, and use multiple investigators to promote reproducibility and minimize bias. Once optimal prompt engineering strategies are identified, efforts should be made to develop guidelines on the safe and effective use of ChatGPT as an orthopedic clinical support tool.

Lastly, additional research is needed to compare the accuracy and reliability of other large language model chatbots. Although recent research has shown higher performance from ChatGPT-4 compared to Gemini by Google, Mistral-7B by Mistral AI, and Claude-3 by Anthropic, these findings focused on medical and surgical care and were limited to recommendations surrounding rotator cuff and anterior cruciate ligament injuries.^6^ Additional research is needed to compare the performance of large language model chatbots across health conditions, asking questions specific to orthopedic and sports physical therapy.

## Conclusion

This Viewpoint asked, “*Is the problem with ChatGPT or the way we use it?*” While several studies challenge the accuracy of ChatGPT,^1,2,4,6,8^ we argue that the answer to this question is still unknown. By systematically evaluating and refining prompting strategies, future research can contribute to more accurate and reliable AI-assisted decision-making in orthopedics, leveraging a technology that has the potential to enhance patient care and clinical outcomes.

## Figures and Tables

**TABLE:** A 2×2 Factorial Design Assessing the Impact of Capacity/Role and Insight on ChatGPT’s Accuracy

	No Capacity/Role	Added Capacity/Role
**No Insight**	No capacity/role is added to the prompt. No insight is added to the prompt. Prompt: “Should electrotherapies (such as TENS/PENS/interferential therapy) be used in the management of nonspecific low back pain and sciatica?”^2(p224)^	“You are an expert physical therapist” is added to the prompt. No insight is added to the prompt. Prompt: “**You are an expert physical therapist.** Should electrotherapies (such as TENS/PENS/interferential therapy) be used in the management of nonspecific low back pain and sciatica?”^2^
**Added Insight**	No capacity/role is added to the prompt. “Use clinical practice guidelines” is added to the prompt. Prompt: “Should electrotherapies (such as TENS/PENS/interferential therapy) be used in the management of nonspecific low back pain and sciatica? **Use clinical practice guidelines.**”^2^	“You are an expert physical therapist” is added to the prompt. “Use clinical practice guidelines” is added to the prompt. Prompt: “**You are an expert physical therapist.** Should electrotherapies (such as TENS/PENS/interferential therapy) be used in the management of nonspecific low back pain and sciatica? **Use clinical practice guidelines.**”^2^

Abbreviations: PENS, percutaneous electrical nerve stimulation; TENS, transcutaneous electrical nerve stimulation.

## Data Availability

There are no data available for this Viewpoint.

## Key Points

- Orthopedic research on ChatGPT often lacks structured prompt engineering, resulting in variable reports of accuracy.
- Frameworks, like CRISPE, can help structure prompts for clinicians, researchers, trainees, and patients.
- Systematic research on prompt frameworks may help to advance ChatGPT as an orthopedic clinical support tool.
